# Pathway-based outlier method reveals heterogeneous genomic structure of autism in blood transcriptome

**DOI:** 10.1186/1755-8794-6-34

**Published:** 2013-09-24

**Authors:** Malcolm G Campbell, Isaac S Kohane, Sek Won Kong

**Affiliations:** 1 Center for Biomedical Informatics, Harvard Medical School, 10 Shattuck Street, Boston, MA 02115, USA; 2 Informatics Program, Children’s Hospital Boston, 300 Longwood Avenue, Boston, MA 02115, USA; 3 Current address: Stanford Neuroscience Program, Stanford University, Stanford CA 94305, USA

**Keywords:** Autism spectrum disorder, Blood gene expression, Nervous system development, Outliers, Pathways

## Abstract

**Background:**

Decades of research strongly suggest that the genetic etiology of autism spectrum disorders (ASDs) is heterogeneous. However, most published studies focus on group differences between cases and controls. In contrast, we hypothesized that the heterogeneity of the disorder could be characterized by identifying pathways for which individuals are outliers rather than pathways representative of shared group differences of the ASD diagnosis.

**Methods:**

Two previously published blood gene expression data sets – the Translational Genetics Research Institute (TGen) dataset (70 cases and 60 unrelated controls) and the Simons Simplex Consortium (Simons) dataset (221 probands and 191 unaffected family members) – were analyzed. All individuals of each dataset were projected to biological pathways, and each sample’s Mahalanobis distance from a pooled centroid was calculated to compare the number of case and control outliers for each pathway.

**Results:**

Analysis of a set of blood gene expression profiles from 70 ASD and 60 unrelated controls revealed three pathways whose outliers were significantly overrepresented in the ASD cases: neuron development including axonogenesis and neurite development (29% of ASD, 3% of control), nitric oxide signaling (29%, 3%), and skeletal development (27%, 3%). Overall, 50% of cases and 8% of controls were outliers in one of these three pathways, which could not be identified using group comparison or gene-level outlier methods. In an independently collected data set consisting of 221 ASD and 191 unaffected family members, outliers in the neurogenesis pathway were heavily biased towards cases (20.8% of ASD, 12.0% of control). Interestingly, neurogenesis outliers were more common among unaffected family members (Simons) than unrelated controls (TGen), but the statistical significance of this effect was marginal (Chi squared *P* < 0.09).

**Conclusions:**

Unlike group difference approaches, our analysis identified the samples within the case and control groups that manifested each expression signal, and showed that outlier groups were distinct for each implicated pathway. Moreover, our results suggest that by seeking heterogeneity, pathway-based outlier analysis can reveal expression signals that are not apparent when considering only shared group differences.

## Background

The study of complex human disorders—diseases influenced by many genetic and environmental factors, such as cancer, diabetes, and autism—has intensified with the rise of sequencing technology, but the majority of genetic heritability remains unaccounted for in most cases [1]. McClellan and King suggested that heterogeneity is the source of this gap, positing that the genetic component of complex disease is in fact a collection of rare or private conditions [2]. Moreover, they asserted that “causality in this context can almost never be resolved by large-scale association or case–control studies”. It is possible that genetic heterogeneity converges onto a final common pathway for each disease, but even at this level there could be one or more related pathways contributing to pathogenesis, with each pathway implicated in only a subset of patients (for example, [3]). This recognition has led to several pathway-based classifications of cancer [4,5].

It is widely accepted that autism spectrum disorders (ASDs) are heterogeneous both phenotypically—for example, among people with ASDs there is much variation in the three core domains of language, social interaction, and range of interests—and in terms of genetic variation [6]. However, most of the published analyses focus on group differences, such as in numbers of genic copy number variations (CNVs) [7-13] and gene expression in genes and pathways [14-22]. By their nature, these methods will blur or collapse the heterogeneity within the ASD group that many have posited characterize this spectrum disorder. Here we have chosen to characterize the heterogeneity of the disorder by finding those cases that are most unambiguously different from other subjects, those that are outliers in one or more gene expression pathways. Specifically, we develop an outlier approach to expression data analysis that searches for pathways in which outliers are biased towards either case or control.

Hawkins defined an outlier to be “an observation which deviates so much from the other observations as to arouse suspicions that it was generated by a different mechanism” [23]. The many methods that have been developed to identify outliers can be grouped into several broad categories [24]. Global methods define outliers using the entire data set as a reference set, whereas local methods use only a subset of samples as reference. Based on the assumption that control samples could be outliers in some pathways and that most case samples will not be outliers in any given pathway, we chose a global outlier method. Labeling methods classify samples as “outlier” or “non-outlier”, while scoring methods assign a continuous outlier score to each sample. In order to assign samples to pathway-specific subgroups, we chose a labeling method. Techniques can also be classified by the properties of the underlying model. The most basic approach is to use statistical tests to calculate a probability of observing a data point given a null distribution, and then apply a threshold to label outliers. More sophisticated approaches include depth- [25], deviation- [26], distance- [27], and density- [28,29] based models.

The Mahalanobis distance is among the most basic techniques for outlier detection, with applications in wide-ranging fields such as the identification of defective machine parts [30], face recognition [31], and cyber security [32]. An analogue of Euclidean distance that scales and centers the data, the Mahalanobis distance gives more weight to variation in directions with lower variance (see Methods). Here we apply it to multivariate transcriptomic data to identify subgroups of outlier samples with distinct, pathway-specific gene expression signatures. Our hypothesis is that outlier samples have gene expression values that were generated by a different mechanism than the rest of samples because of genomic variants in the pathway’s genes or in their regulators. A common problem with outlier-based methods is that outliers can strongly influence estimates of the parameters of the normal data. We address this issue by employing the robust minimum covariance determinant to estimate mean and scatter [30,33].

Many applications of outlier-methods to genomic data have already been developed, primarily to identify subsets of tumor samples with different chromosomal translocations or activated oncogenes [34-41]. These methods propose various ways of defining a cutoff between outlier and non-outlier samples for a single gene, motivated by the fact that different oncogenes may be activated in different tumor subtypes, leading to outlier expression for these genes in some samples but not others [34-38,40]. Recently, Luo and colleagues brought outlier methods to autism research [42]. By integrating transcriptomic and copy number variation (CNV) data from autistic patients and controls, they linked genes with outlier expression values in cases to specific CNVs. While powerful, all of these methods search for outliers at the level of the gene. The approach taken here instead searches for outliers within the multidimensional space corresponding to the genes in a pathway, based on the hypothesis that these pathways allow the identification of shared endophenotype [43].

We applied our method to an autism data set derived from peripheral blood with 70 ASD and 60 control samples, collected by the Translational Genomics Research Institute (TGen) in Pheonix, Arizona, which we refer to as the TGen data set. The analysis revealed three pathways—neuron development, nitric oxide (NO) signaling, and skeletal development—with significantly more case outliers than control outliers according to Fisher’s exact test. Analysis of a second data set, consisting of 221 ASD and 191 control samples and referred to hereafter as the Simons data set, confirmed that neurogenesis was perturbed in a subset of samples. In this data set, controls were unaffected family members (188 siblings and 3 mothers), and the proportion of control neurogenesis outliers was higher than in the TGen data set where the controls were unrelated to the cases. Conventional differential expression of pathways that use overall group differences (e.g. GSEA) did not identify the perturbation of the above pathways as significant.

## Results

Our aim was to characterize samples as “outliers” or “non-outliers” in prior-knowledge based pathways, with the hypothesis that, within a pathway, outliers represent samples that are biologically perturbed. For analysis, we collected 2,159 pathways that included modified Gene Ontology (GO) terms [44], Kyoto Encyclopedia of Genes and Genomes (KEGG) pathways [45,46], Reactome pathways [47], and Biocarta pathways (http://www.biocarta.com) from the Molecular Signatures Database (MSigDb) version 3.0 [48], two genesets consisting of differentially expressed genes for autism-linked syndromes i.e., Fragile X mental retardation and 15q duplication based on the data of Nishimura and colleagues [19], and two sets of *de novo* mutation-containing genes from two recent exome sequencing studies [49,50] (see Methods).

Although each of these pathways consists of 10 to 300 genes, we reduced these many dimensions into a single quantitative measure for each sample. First, we applied principal component analysis (PCA) to the multidimensional space of genes in the pathway and retained the principal components that accounted for 90% of variance [51]. In the TGen data set, the median number of retained principal components was 6 (IQR = 4–9), whereas in Simons this number was larger (26, IQR = 17–47), a difference that can be at least partially explained by the difference in sample size. After projecting the data into PCA space, we represented each sample by a Mahalanobis distance to the centroid of all samples [30,52,53]. In theory, these distances follow a square root chi-squared distribution under the null hypothesis. This allowed us to define pathway-specific outliers based on the theoretical chi-squared 97.5^th^ percentile, which corresponds to a p-value < 0.025 for a one-sided test [30]. Having categorized the samples into outliers and non-outliers in each pathway with this threshold, we then searched for pathways where the outliers were significantly biased towards either case or control.

### Identification of outlier-enriched pathways

In the TGen data set, we initially found five pathways enriched for case outliers at FDR < 10%. No pathway was enriched for control outliers at this threshold. The genesets characterizing 15q duplication and Fragile X mental retardation were not enriched for outliers in our data set, nor were the sets of genes that contained *de novo* mutations in two recent studies [49,50]. The case-enriched pathways were axonogenesis (GO:0007409, modified by MSigDb), neurite development (GO:0031175, modified by MSigDb), neuron development (GO:0048666, modified by MSigDb), nitric oxide (NO) signaling pathway (Biocarta), and skeletal development (GO:0001501, modified by MSigDb). To check for the confounding effect of age, we performed propensity sampling (see Methods). Briefly, propensity sampling selects subsets of cases and controls that are matched for age and repeats the procedure on this reduced data set. All five pathways ranked highly after propensity sampling for age (ranks 2, 16, 7, 18, and 3 out of 2,159 pathways, respectively) indicating that age was not an important confounder. The complete results from propensity sampling, reported as average p-values across 100 trials, are included as Additional file 1. P-values were less significant after propensity sampling because of iterations in which outliers were excluded.

Among the five pathways that were significantly enriched with case outliers, axonogenesis, neurite development, and neuron development are highly redundant genesets: axonogenesis is contained within neurite development, which is contained within neuron development. The size of these genesets is 43, 53, and 61 genes, respectively. Because they are almost identical, these three pathways captured a very similar signal. As expected, the Mahalanobis distance distributions for these pathways were highly correlated (Kendall’s tau ≥ 0.74 for all three pairs, *P* < 6.86 × 10^− 4^. Therefore, we selected only the largest pathway, neuron development, for further analysis. In contrast, neuron development shares only one gene with skeletal development (*GLI2*) and none with NO signaling. NO signaling and skeletal development do not share any genes, and the correlation of their Mahalanobis distance distributions was not significant compared to the distribution of Kendall’s tau correlation for all pairs of pathways (Kendall’s tau = 0.29, *P* = 0.349). For these reasons, we selected neuron development, nitric oxide signaling, and skeletal development as the candidate pathways in which a subgroup of patients was detected as outliers in TGen. Table 1 enumerates the number of case and control outliers in these pathways along with their Fisher’s exact test p-values. For reference, in the average pathway, 10.7 cases (15.3%) and 8.4 controls (14.0%) were outliers.

**Table 1:** Case/control outlier counts in outlier-enriched TGen pathways

**Pathway**	**Case outliers**	**Control outliers**	**Fisher’s exact test P**
Neuron development	20 / 70 (28.6%)	2 / 60 (3.3%)	9.97 × 10^− 5^
Nitric oxide signaling	20 / 70 (28.6%)	2 / 60 (3.3%)	9.97 × 10^− 5^
Skeletal development	19 / 70 (27.1%)	2 / 60 (3.3%)	2.03 × 10^− 4^
**Total**	**35 / 70 (50%)**	**5 / 60 (8.3%)**	1.47 × 10^− 7^

**Figure 1:**
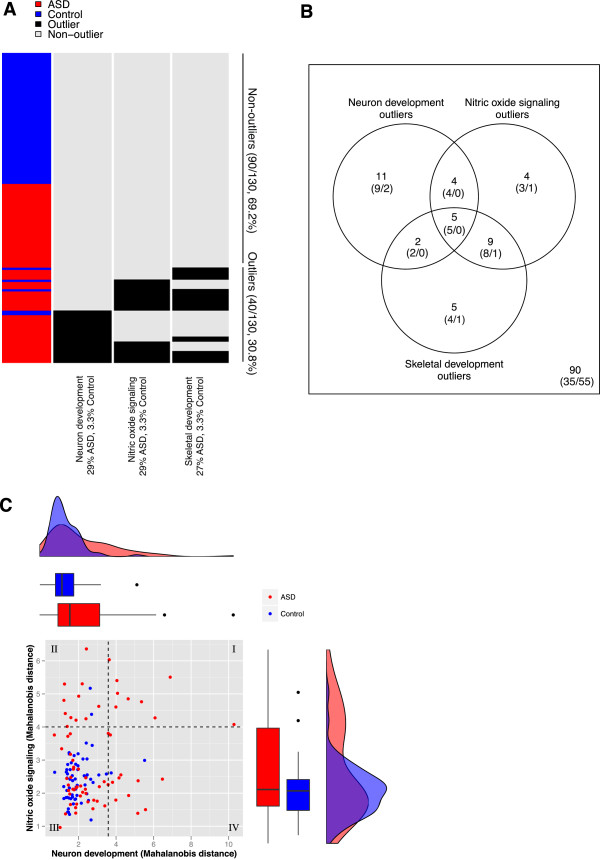
**Outlier-enriched pathways in the TGen data set. A)** Heatmap marking outliers for each outlier-enriched pathway: neuron development, nitric oxide (NO) signaling, and skeletal development (Figure 4, step 4). Samples sorted by outlier status followed by diagnosis. **B)** Venn diagram showing the overlap of outlier samples for the three pathways in Figure 1A. The numbers of case and control outliers, respectively, are shown in parentheses. **C)** A comparison of Mahalanobis distance values (Figure 4, step 2) in neuron development and NO signaling. Marginal density plots show the distributions of case (red) and control (blue) samples.

This produced a clustering of samples into overlapping subgroups where each subgroup consisted of the outliers in a candidate pathway (Figure 1A). Overall, 30.8% (40/130) of samples were outliers in at least one pathway. The overlap of these pathway-specific outlier groups is shown in Figure 1B. The highest overlap was between NO signaling and skeletal development, with 14 samples in both groups out of 22 NO signaling and 21 skeletal development outliers. Remarkably, the five samples that were outliers in all three pathways were all cases, and only one control was an outlier in more than one pathway (NO signaling and skeletal development). Figure 1C shows a more detailed comparison of two candidate pathways, neuron development and NO signaling. Nine samples were outliers in both pathways (quadrant I), 13 samples (11 ASD and 2 controls) were outliers in NO signaling but not neuron development (quadrant II), and 13 samples (11 ASD and 2 controls) were outliers in neuron development but not NO signaling (quadrant IV). A 3-dimensional PCA plot of the neuron development pathway, revealing the multivariate structure behind the outlier calculation, is shown in Additional file 2.

### Differential expression analysis

**Figure 2:**
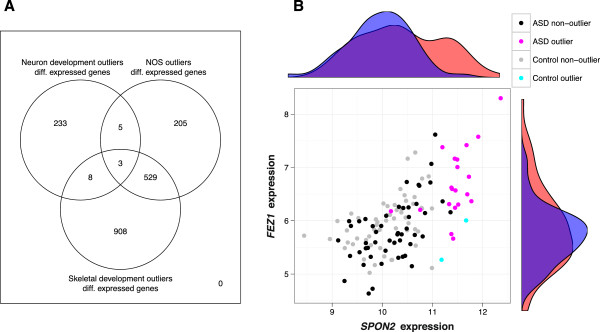
**TGen outlier-group differentially expressed genes (Figure **4**, step 5).** Differential expression was calculated using t-tests on log (base 2) expression values at FDR < 5%. **A)** Overlap of differentially expressed genes for the three outlier groups. **B)** Comparison of the log (base 2) expression levels of the two most differentially expressed genes in the neuron development pathway, *SPON2* and *FEZ1*. Marginal density plots show the distributions of case (red) and control (blue) samples.

We opted to determine the leading edge genes that distinguished each outlier group from the rest of samples. To do so, we performed standard differential expression analysis of outliers vs. non-outliers in the three candidate pathways (see Methods). There were 249 differentially expressed genes for neuron development (22 outliers vs. 108 non-outliers), 742 for nitric oxide signaling (22 vs. 108) and 1448 for skeletal development (21 vs. 109) at the same FDR < 5% (Figure 2A). These included 8, 14, and 26 known autism candidate genes respectively from the Simons Foundation Autism Research Initiative (SFARI) Gene 2.0 database [54], which contained 369 genes as of July, 2012 (Table 2). Based on hypergeometric tests, this overlap was significant for the neuron development group, but not for the other two (*P* = 0.0310, 0.314, 0.708 respectively). Because of the relatively large number of samples that were outliers in both NO signaling and skeletal development (Figure 1B), the differentially expressed genes for these two subgroups were highly overlapping.

**Table 2:** Differentially expressed autism candidate genes for each pathway subgroup

**Neuron development**			**Nitric oxide signaling**			**Skeletal development**		
**Gene**	**log2(FC)**	**q-value**	**Gene**	**log2(FC)**	**q-value**	**Gene**	**log2(FC)**	**q-value**
*BZRAP1*	0.759	7.39e-08	***SYNGAP1***	0.760	0.000173	***SYNGAP1***	0.769	4.24e-06
*CD38*	0.645	0.0270	*GPC6*	0.510	0.0437	***KCTD13***	0.564	9.09e-07
*PDE4A*	0.430	0.00711	***KCTD13***	0.497	0.000921	***NSD1***	0.492	6.91e-08
*AUTS2*	0.387	0.00217	***DMPK***	0.391	0.00565	*PCDHGA11*	0.485	2.88e-06
*ADRB2*	0.363	0.00122	***NSD1***	0.340	0.00847	***DMPK***	0.478	1.24e-05
*ADA*	0.330	0.00246	*CACNA1G*	0.285	0.0494	*NCKAP5L*	0.374	0.000301
*STK39*	0.306	0.0210	***MED12***	0.215	0.0184	*SATB2*	0.330	0.0169
*BCL2*	-0.235	0.0141	***CD44***	0.164	0.0276	*NOS2A*	0.327	0.0102
			*TSN*	-0.172	0.0127	*DPP6*	0.325	0.0382
			***PEX7***	-0.228	0.0283	*EPHB6*	0.308	0.00248
			*TMLHE*	-0.245	0.0461	***MED12***	0.305	7.87e-05
			*EPHA6*	-0.287	0.0368	*NRP2*	0.290	0.0302
			*DIAPH3*	-0.510	0.0247	*TSC2*	0.238	0.0352
			***TPH2***	-0.548	0.0183	*MAPK3*	0.220	0.0283
						*DRD2*	0.179	0.0316
						*RIMS3*	0.177	0.0289
						***CD44***	0.151	0.0301
						*RPL10*	-0.102	0.0181
						*ADSL*	-0.155	0.0440
						*SLC25A12*	-0.183	0.0431
						*ARHGAP15*	-0.187	0.0271
						*DUSP22*	-0.194	0.0204
						***PEX7***	-0.212	0.0408
						*RORA*	-0.266	0.0295
						*EIF4E*	-0.343	0.0293
						***TPH2***	-0.822	0.00313

Genes in bold were differentially expressed in both nitric oxide signaling and skeletal development outliers, which were the groups with the highest overlap (Figure 1B).

The top differentially expressed gene for the neuron development group was *SPON2*, which codes for spondin-2, an extracellular matrix protein. Also known as M-spondin and mindin, spondin-2 has been shown to direct the growth and adhesion of embryonic hippocampal neurons in a rat model [55]. As an extracellular matrix protein involved in neuronal cell adhesion, it plays a role one of the emergent themes of ASD neurobiology [56]. The only other gene in the intersection neuron development with the 249 differentially expressed genes was *FEZ1*, which codes for fasciculation and elongation protein 1. *FEZ1* was recently shown to interact with *DISC1*, a known candidate gene for both autism and schizophrenia [57,58]. Expression levels of *SPON2* and *FEZ1* are plotted against each other in Figure 2B, showing that most neuron development outlier samples had extreme values in one or both of these genes. However, some non-outlier samples had extreme expression values in these genes and vice versa, indicating that the pathway structure is also important. A complete list of differentially expressed genes for the three pathways is provided in Additional file 3.

### Comparison with group difference tests

To check whether these candidate pathways could be identified by group comparison methods, rendering this outlier-based approach irrelevant, we performed standard differential expression followed by hypergeometric tests for enrichment among the MSigDB pathways (see Methods). Using this approach, 437 genes were differentially expressed at FDR < 5%, 5 pathways were identified at FDR < 5%, and 45 pathways were identified at FDR < 10% (Additional file 4). It is likely that the significance of these hypergeometric tests was inflated by our filtering for robustly expressed genes, which are biased towards genes that are part of known pathways. Nevertheless, none of neuron development, NO signaling, or skeletal development was significant according to this analysis, with q-values of 0.365, 1, and 1, respectively. Of the pathways that were significantly enriched with differentially expressed genes by hypergeometric tests, only the GO term axon guidance (GO:0007411, modified by MSigDB), a 22-gene pathway that is entirely contained within neuron development, was equivalent to or contained within one of the three outlier pathways (Additional file 4). Axon guidance contained 4 differentially expressed genes (hypergeometric *P* = 0.000944, q-value = 0.0702); these genes were *SPON2*, *SIAH1*, *SLIT1*, and *FEZ2*. Interestingly, in the group difference comparison, *FEZ2* and not *FEZ1* was differentially expressed. Since axon guidance was one of 45 pathways identified at FDR < 10% using hypergeometric tests whereas neuron development was one of 3 pathways identified at FDR < 10% using the outlier method, we conclude that our method identifies this signal with greater specificity. The three outlier-enriched pathways were not identified as significantly up or down regulated in cases using GSEA even at FDR < 25%. Indeed, no pathways were significant at FDR < 10% by GSEA. Taken together, these data indicate that our outlier method captured a signal that was not evident at the group difference level.

### Comparison with gene-level outlier analysis

To determine whether pathway-level analysis held any advantage over gene-level analysis, we performed gene-level outlier tests (see Methods). Our gene-level analysis was analogous to our pathway-level analysis in that we defined outliers based on a hard threshold and then performed Fisher’s exact tests to compute outlier enrichment. 822 genes were significant at FDR < 5% (Additional file 5). 14 MSigDB pathways were significant at FDR < 10% by hypergeometric tests (Additional file 5). Neuron development, NO signaling, and skeletal development were not among them. Out of these 14 pathways, only negative regulation of developmental process (GO:0051093, modified by MSigDB) overlapped significantly with any of the three outlier-enriched pathways (namely, neuron development and skeletal development; see Additional file 5 for overlap statistics), but the overlap was not nearly as significant as a complete containment, as in the case of neurogenesis and neuron development. Interestingly, *SPON2* was the fifth ranked outlier gene (*P* = 4.83 × 10^− 5^, q-value =0.0118), and *FEZ1* was also significant (*P* = 0.0138, q-value = 0.0392), but neuron development was not significantly overrepresented in the 822 outlier genes overall. This suggests that the subtle contributions of other genes in the neuron development pathway enabled pathway-level analysis to capture a signal that was lost at the gene-level.

### Validation in an independent data set

For validation, we applied the same analyses to the Simons data set. No genes were differentially expressed (minimum FDR q-value = 1) and no pathways were differentially expressed at FDR < 25% using GSEA. Using the outlier method presented here, no pathways were significant at FDR < 10%, and all pathways with uncorrected Fisher’s test p-value < 0.05 are listed in Additional file 6. The highest ranked outlier-enriched pathway was the bone remodeling RANKL pathway (Biocarta). In this pathway, we identified 39 / 221 (17.6%) of cases and 14 / 191 (7.3%) of controls as outliers (Fisher’s exact test *P* = 0.00185). The RANKL pathway regulates bone homeostasis [59,60], but since it shares no genes with the MSigDb skeletal development pathway, a comparison of the RANKL signal in Simons to the skeletal development signal in TGen is purely speculative.

**Figure 3:**
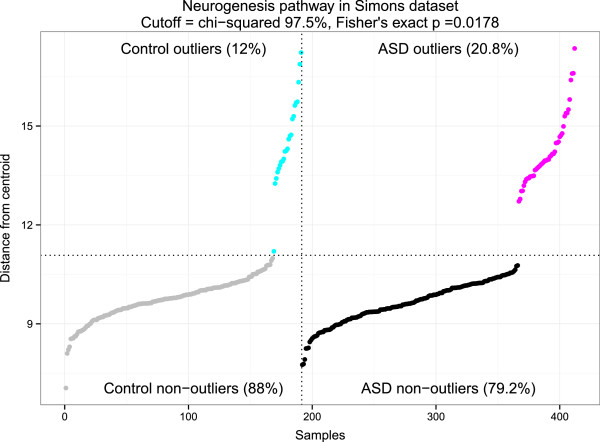
**Neurogenesis outliers in the Simons data set.** The y-axis is in units of Mahalanobis distance from the pooled centroid of cases and controls, and the x-axis is samples. One control sample was misclassified as an outlier due to the choice of $\chi_{0.975}^{2}$ as the hard threshold.

None of the three TGen candidate pathways were initially identified as outlier-enriched in the Simons data set with Fisher’s exact test p-values of 0.204 (neuron development), 1.0 (NO signaling), and 0.703 (skeletal development) and case/control outlier percentages of 20.8%/15.7%, 16.7%/12.6%, and 17.6%/19.4%, respectively. These numbers were fairly typical given average outlier percentages of 16.6% for cases and 15.8% for controls. However, we found that neurogenesis (GO:0022008, modified by MSigDB), a 93-gene pathway that contains all 61 neuron development genes, ranked highly among the pathways biased towards ASD outliers. Remarkably, neurogenesis ranked 9^th^ out of 2,159 pathways for case-specific outlier enrichment (99.5^th^ percentile), and 13^th^ for two-sided tests (99.3^rd^ percentile). As shown in Figure 3, we identified 46 / 221 (20.8%) of cases and 23 / 191 (12.0%) of controls as outliers (Fisher’s exact *P* = 0.0178). While neurogenesis did not pass the FDR < 10% significance threshold based on the multiple hypothesis-corrected Fisher’s test (indeed, no pathways passed this threshold in the Simons data set), we consider this rank-based evidence noteworthy given the statistical significance of the pathway in the TGen data set. One control sample that clearly belongs to the non-outlier group was called an outlier, but was very close to the outlier threshold. Nevertheless, neurogenesis was highly ranked despite this classification error, which weakened the result. Interestingly, a greater proportion of controls had a neurogenesis signature in the Simons data set than in the TGen data set (12% vs. 3%, chi-squared *P* = 0.0858), which is consistent with the fact that the Simons controls were unaffected family members rather than unrelated children. Out of 46 case outliers and 23 control outliers, there were six proband/sibling pairs, of which two were sex matched.

Differential expression analysis revealed the genes that drove the clustering of the neurogenesis subgroup in the Simons data set. At FDR < 5%, 1,969 genes were differentially expressed between neurogenesis outliers and all other samples (Additional file 7). Of these, 13 were among the 93 genes in the neurogenesis pathway. Three out of these 13 neurogenesis genes were known autism candidate genes according to the SFARI database: *NRXN3, ROBO1,* and *NRCAM*. All three were overexpressed in the neurogenesis outlier subgroup. Moreover, out of the total 1969 differentially expressed genes, 35 were found in the SFARI database. Interestingly, *SPON2*, the most significant neuron development gene in TGen, was marginally differentially expressed in the Simons neurogenesis group (*P* = 0.0889, q-value = 0.172).

Because the Simons data set consisted largely of pairs of siblings, we performed an alternate analysis for this data set using McNemar’s test instead of Fisher’s test. There were 168 proband/sibling pairs out of 412 samples. On the reduced data set consisting of these 168 pairs of siblings, no pathways were significant: all pathways had q-values of 1 (see Additional file 8 for McNemar’s test results for all 2,158 pathways). In the neurogenesis pathway, 19% of ASD samples were identified as outliers versus 13.1% of controls, yielding a McNemar’s test p-value of 0.155. There were seven sibling pairs (4.2% of both cases and controls) in which both siblings were outliers, which is greater than expected by chance but not significant by hypergeometric test (*P* = 0.0934). Because the direction of the effect remained the same but was less significant, and because Fisher’s test p-values were highly correlated with McNemar’s test p-values on the reduced data set (*ρ* = 0.864), we attribute the loss of significance to the elimination of 76 samples and the resulting loss in power, rather than the use of an alternate method. Indeed, this result is consistent with the interpretation that the neurogenesis signal is enriched among siblings of probands as compared to unrelated controls.

## Discussion

ASD, like other complex disorders such as diabetes and heart disease, is almost certainly associated with the effect of multiple genes as well as environmental factors. To date, no more than 20% of cases have been linked to structural genomic variants such as *de novo* CNVs and mutations, and monogenic syndromic disorders. To further understand the heterogeneity of ASD genetic architecture reflected in the blood transcriptome, we developed a novel approach using outlier statistics. To demonstrate the plausibility, we used two independently collected data sets of ASD and controls. Only ~30% of cases shared molecular signatures including neural development (29% of cases), NO signaling pathway (29% of cases), and skeletal development (27% of cases). These pathways could not be identified with group comparison or gene-level outlier methods, and the significantly perturbed cases for these pathways were not identical. Overall, our approached identified 50% of cases but only 8% of controls as outliers in at least one of these pathways.

To date, most emergent biological themes in ASDs have fallen into one of three categories: neuroanatomical, systems, and molecular and cellular [56]. Neuroanatomical observations of altered brain growth patterns [61-63] and minicolumnopathy [64] are the most reproducible clinical signatures of ASDs. Pathways affecting cellular proliferation such as the PI3K-AKT-mTOR pathway have been hypothesized to affect abnormal brain growth in ASD, but no concrete link between such pathways and brain growth patterns exists as yet [56]. At the systems level, evidence has accumulated for functional alterations in white matter tracts [65-68] and overall imbalance between excitation and inhibition in the brain [69-72]. Cellular and molecular themes have converged on the function and structure of the synapse. Rare or *de novo*, deleterious mutations were found in ionotropic glutamate receptors [49,73], voltage-gated sodium channels [49,50,74], and voltage-gated calcium channels [75,76]. Neurexins and neuroligins are involved in neuronal adhesion and have been heavily implicated in ASDs by cytogenetic analysis [77], CNV studies [8,9,11,49] and knockout mouse models [70,72,78]. Similarly, candidate genes *SHANK2* and *SHANK3* code for scaffold proteins in the postsynaptic density. Other ASD candidate genes with protein products in the postsynaptic density include *FMR1* and associated genes *MET*, *PTEN*, *TSC1*, *TSC2*, and *NF1*, all of which are involved in translation, as well as genes involved in protein degradation such as *UBE3A*, *PARK2*, *RFWD2*, *FBXO40*, and *USP7*[56]. In summary, anatomical, physiological, mouse-model, and human genetic studies have implicated brain growth, white matter connectivity, synaptic transmission, and the structure of the synapse as promising biological themes in ASD.

In this context, our discovery of three pathways related to neural development—axonogenesis, neurite development, and neuron development, which we collapsed together for analysis—was notable as defects in early neurodevelopmental processes such as neuronal survival, differentiation, migration and synaptogenesis may cause neurobiological abnormalities in ASD [79]. The NO signaling pathway contains genes involved in the glutamate NMDA receptor, as well as in the calcium/calmodulin and NO mediated second messenger systems that regulate long-term potentiation and other activity dependent developmental processes. Moreover, neurogenesis was dysregulated in a subgroup of cases from an independently collected cohort. Specifically, in the Simons data set we identified 20.8% of cases and 12.0% of unaffected family members as neurogenesis outliers.

Outlier samples also showed gene-level differences compared to non-outlier samples. By comparing outliers and non-outliers, we could identify differentially expressed genes that were specific to outlier subgroups. Among these, *FEZ1* was recently shown to interact with *DISC1*, a susceptibility gene for schizophrenia and other mental disorders [57]. In that paper, the authors show that fasciculation and elongation protein zeta-1 (FEZ1) acts together with Disrupted-in Schizophrenia 1 (DISC1) to regulate dendritic growth in the hippocampus of adult mice. Interestingly, *DISC1* is also an ASD candidate gene; variation in *DISC1,* located at 1q42, was correlated with autism in a Finnish cohort [58]. While *FEZ1* was not differentially expressed between cases and controls overall in the TGen data set (*P* = 0.238), we were able to detect differential expression of *FEZ1* in a subset of cases using our heterogeneity-based approach (*P* = 1.85 × 10^− 7^, q-value = 5.95 × 10^5^). Similarly, *SPON2*, whose protein product spondin-2 was shown to direct the development of hippocampal neurons in rats [55], was highly over-expressed in neuron development outliers (*P* = 2.29 × 10^− 19^, q-value = 4.48 × 10^− 15^, differential expression rank = 1/21,184) but this significance was diluted at the group difference level (*P* = 0.000946, q-value = 0.0430, differential expression rank = 292/21,184). Interestingly, we could recover most outlier cases from the distributions of *SPON2* and *FEZ1* alone (Figure 2B). While gene-level analysis detected these two genes at FDR < 5%, neuron development was not overrepresented among the outlier genes overall, indicating that other genes in the pathway also played an important role.

Our method and the two data sets used in our study had several limitations. Due to their incompleteness and generality, the pathway definitions from MSigDB imperfectly describe the underlying biology of ASD. Nevertheless, we chose to use these definitions as opposed to data-driven pathways to avoid over-fitting. Clinical definitions of ASD are constantly changing, and include a broad swath of individuals with heterogeneous disorders; while this was the motivation for our analysis, it is also conceivable that misdiagnosis due to overly inclusive criteria led to the inclusion of false-positive outliers in our study. It is possible that genetically distinct cohorts were recruited for the two data sets, as samples were collected at two geographically distant study sites with different local ancestral structures. Although we tried to reduce technical variation such as batch effects in each data set, it is possible that some technical artifacts remained. There will also inevitably be technical variability between two genomic profiling facilities and microarray platforms. Therefore, it is unsurprising that we were not able to replicate all of our results: specifically, we were unable to identify NO signaling and skeletal development signatures in the Simons cohort, and the RANKL pathway, while perhaps related to skeletal development, was the top-ranking outlier-enriched pathway in the Simons data set but not significantly outlier-enriched the TGen data set. Finally, because we used blood gene expression profiles as a surrogate for studying genomic alterations in a neurodevelopmental disorder, the difference in transcriptomic repertoire between blood and brain might have limited us to characterizing only 50% of samples in our results. Because of these limitations, this study and its results should be considered exploratory, showing the potential benefits of a novel approach, but not conclusive.

A large number of samples from different cohorts and the integration of genetic and transcriptomic profiles are essential for the identification of subgroups that may share clinical features, treatment responses, and prognostic characteristics [80]. Along with the alarming increase in ASD prevalence in the last few decades has come an accumulation of genetic and genomic profiling data [42,49,50,74,81], and yet the group difference between ASD and non-ASD is not obvious by any measure. We characterized 50% of cases with specific genomic signatures using an outlier-based approach, which will be strengthened by the integration of different modalities of genomic data such as whole-genome and whole-exome sequences. Looking farther into the future, true personalized medicine will only be achieved when individual genetic and genomic characteristics are combined with clinical and other phenotypic information.

## Conclusions

In this study, we applied a novel, pathway-based outlier method to two publicly available autism gene expression data sets from peripheral blood (TGen and Simons). Analysis of the TGen data set revealed three non-identical subgroups of samples with perturbations in the neuron development, nitric oxide signaling, and skeletal development pathways. In the Simons data set, we also found a subset of patients with a perturbed neurogenesis signature, but were unable to convincingly replicate nitric oxide signaling or skeletal development. A greater proportion of unaffected family members (Simons controls) manifested a neurogenesis signature than did unrelated children (TGen controls), possibly due to the shared genetic background between probands and their family members. While pathway-based classifications of cancer have been developed, this is both the first application of such methods to ASDs, and the first integration of pathway-based classification with outlier methods. These results show that pathway-based outlier analysis is useful for the study of complex disorders, and add to the growing body of evidence that peripheral blood gene expression data contain useful markers for neurodevelopmental disorders.

## Methods

### Gene expression data sets

We analyzed two previously published blood gene expression data sets. The first data set was from the Translational Genetics Research Institute (TGen), and consisted of 144 CEL files from Affymetrix HG-U133 Plus 2.0 chips (78 from ASD samples and 66 from controls). Quality control left 130 arrays (70 case and 60 control). This data set is available on the Gene Expression Omnibus (GEO) as GSE25507 [82]. The second data set was from the Simons Simplex Consortium (SSC), and consisted of 439 Illumina Whole Human Genome Array Human REF-8 version 3.0 arrays (233 case and 206 control). After quality control, 412 samples remained (221 case and 191 control). The 191 controls consisted of 188 unaffected siblings and 3 mothers. All 3 mothers had a child among the probands, and there were 168 proband/sibling pairs. The other 20 controls were unaffected siblings of probands not included in the study. We refer to this data set as the Simons data set; it is available on GEO as GSE37772 [42]. See “Preprocessing” for detailed microarray preprocessing steps. Phenotype information for both data sets is summarized in Additional file 9.

### Genesets

Prior knowledge-based genesets consisting of Entrez Gene Identifiers (Entrez IDs) were downloaded in Gene Matrix Transposed (GMT) format from the Molecular Signatures Database (MSigDB) version 3.0 [48]. Of the available genesets, we used those that are expert-curated, namely C2:CP (canonical pathways), and C5 (modified Gene Ontology term genesets). After filtering out large (>300 genes) and small (<10 genes) genesets, there were 2157 genesets in these categories as of January, 2012. To this we added genesets consisting of differentially expressed genes from the comparison of blood gene expression profiles from patients with Fragile X syndrome and 15q duplication to controls [19], and two sets of *de novo* mutation-containing genes from exome sequencing studies [49,50], resulting in a total of 2161 genesets. These genesets were mapped to probesets on the Affymetrix HG-U133 Plus 2 array using the annotation table from the Affymetrix website dated June 9^th^, 2011. Probesets ending in “x_at” were discarded because this suffix indicates that the probeset may bind to multiple transcripts. In total, these genesets contained 9,347 unique Entrez IDs, 98% of which (9,175) mapped to at least one probeset on the Affymetrix chip, covering 35% of all probesets. Throughout this paper, the terms “geneset” and “pathway” are used interchangeably.

### Preprocessing

TGen samples were quantile normalized and background adjusted with Probe Logarithmic Intensity Error using Affymetrix Power Tools Version 1.14.4 [83]. All 144 chips had high mean inter-array correlation (≥ 0.9). Further array-level quality checks were performed by visually inspecting MA plots from Bioconductor’s AffyPLM package [84]. The TGen data set did not exhibit strong batch effects (Additional file 10), and the case and control groups were balanced for race (Additional file 9). Therefore, we decided not to use batch effect correction such as ComBat [85] because we observed that this can introduce bias to the data. However, we noticed that TGen data set contained a group of samples that were outliers in almost every pathway. These samples also correlated with the first surrogate variable from Surrogate Variable Analysis [86], indicating that they were technical outliers. We defined “total outlier” samples as the top 90^th^ percentile of the mean Mahalanobis distance distribution across all pathways, and removed them to ensure that remaining samples were not array-wide outliers but rather outliers in a specific set of pathways, leaving 130 arrays (70 case and 60 control). See Additional file 10 for PCA plots of the entire TGen data set before and after the removal of total outlier samples.

For preprocessing of the Simons data set, we followed the procedure of the original authors [42]. Based on hierarchical clustering using inter array correlation (IAC) as the distance metric, 27 arrays were discarded, leaving 412 (221 case and 191 control). The remaining samples were quantile normalized, and ComBat was performed with the default parameters to reduce batch effects. We then fit the data set to a linear model with collection batch, sex, and age as independent variables and kept the residual values. See Additional file 10 for PCA plots of the Simons data set before and after ComBat and linear modeling.

### Propensity sampling

To check that our results were not due to the influence of confounders, we performed repeated propensity sampling [87]. There was a significant difference in the age distributions of case and control samples in TGen, with cases being younger in general (see Additional file 9). We performed logistic regression of diagnosis on age, and binned the resulting probabilities into five bins. Then we sampled each bin so that numbers of cases and controls were matched. The resulting data set is said to be “propensity matched”. This procedure was repeated 100 times to include unused samples, and for each iteration the reduced data set was run through the rest of the procedure. The resulting p-values were averaged and compared to p-values generated from the full data set.

### Mahalanobis distance and outlier vs. non-outlier classification using the chi-squared distribution

**Figure 4:**
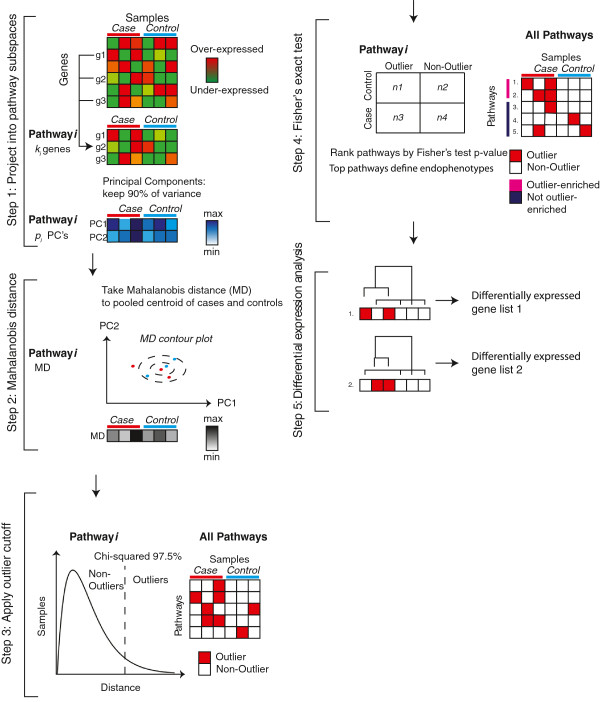
**Analysis flow chart.** In brief, the steps of the method were: **1)** project samples onto the principal components (PC) of pathway subspaces, **2)** calculate the 1-dimensional Mahalanobis distance distribution of samples in the PC space of each pathway, **3)** classify samples as “outliers” or “non-outliers” in each pathway based on the 97.5^th^ percentile of the chi-squared distribution, **4)** employ Fisher’s exact test to identify pathways that are specifically enriched for case or control outliers, using the False Discovery Rate (FDR) q-value of Storey and Tibshirani to correct for multiple hypothesis testing [88], **5)** for each subgroup of samples corresponding to the outliers in a candidate pathway, perform standard differential expression analysis to determine the genes responsible for the grouping.

We used a similar approach as described in Kong et al. to project samples into dimensionally-reduced geneset subspaces, aggregating the signal at the pathway level to improve robustness to gene level noise [53]. In the subset of the expression matrix corresponding to each geneset, we projected the data onto the space of first *n* principal components that account for ≥ 90% of variance (Figure 4 step 1) [51]. For the TGen data set, raw expression levels, as output by PLIER, were input to PCA. For the Simons data set, residual values after linear modeling were used. In each dimensionally-reduced geneset subspace, we calculated each sample’s Mahalanobis distance to the centroid of all samples, using the Fast Minimum Covariance Determinant algorithm to estimate the robust pooled covariance matrix and centroid [30] (Figure 4 step 2). The Mahalanobis distance formula is:

$$D_{M}\left(x\right)=\sqrt{{\left(\chi ‒\mu \right)}^{T}S^{‒1}\left(\chi ‒\mu \right)}$$

where *x* is the sample in question, *S* is the robustly estimated covariance matrix of the pathway subspace, and *μ* is the robust mean vector. Performing the Mahalanobis distance calculation for each pathway produced a distance matrix of pathways by samples. Theoretically, the Mahalanobis distance will follow a chi-squared distribution, with the number of degrees of freedom equaling the number of principal components used to calculate the distance. We defined the outlier cutoff as the standard value $\sqrt{\chi_{d,.975}^{2}}$, the square root of the 97.5^th^ percentile of the chi-squared distribution with *d* degrees of freedom, generating a binary matrix of pathways by samples where 1/0 represents outlier/non-outlier (Figure 4 step 3). One problem with the Mahalanobis distance is that as the degree of freedom increases, samples yield increasingly similar values [24]. We surmount this problem by applying the Mahalanobis distance to a small number of principal components (typically less than 10) rather than the complete multidimensional gene space (10–300 genes per pathway).

### Selection of significant pathways using Fisher’s exact test

Once we had defined which samples were outliers in each pathway, we used Fisher’s exact test to select pathways where outliers were predominantly case or control samples (Figure 4 step 4). Because our tests were symmetric with respect to diagnosis, pathways could be enriched for case or control outliers. The inclusion of control-enriched pathways provided an automatic estimation of the false-positive rate for pathway detection. Fisher’s test p-values were transformed to FDR estimates following the q-value method of Storey and Tibshirani [88]. We defined a significance threshold of FDR < 10% and called any pathway that passed this threshold “outlier-enriched”.

### Pathway-specific differential expression

Differential expression was evaluated for each pathway by comparing outliers in that pathway to the remaining samples (Figure 4 step 5). Multiple probe sets were collapsed to the gene level by taking the maximum expressed probe sets, then Welch’s t-test was then performed on log (base 2) expression values for each gene. We corrected for multiple comparisons using the q-value FDR estimation with a significance threshold of FDR < 5%.

### Group difference comparison

For comparison, we also tested group differences between cases and controls at the pathway level using hypergeometric tests on differentially expressed genes. To calculate differential expression, probes were log (base 2) transformed and collapsed to the gene level by taking the maximum probe for each Entrez gene ID. We filtered for robustly expressed genes by requiring that at least 2/3 of samples had log (base 2) expression > 7, and we performed t tests on the remaining genes with an FDR cutoff of 5%. We then performed hypergeometric tests for enrichment of these genes among the MSigDb pathways. We also performed GSEA on all pathways using Gene Pattern [89] with 1000 sample label permutations, ranking genes by signal to noise ratio.

### Gene-level outlier analysis

We also compared pathway-level outlier analysis to gene-level outlier analysis. The expression set was collapsed to the gene level by taking the maximum probe for each gene. Then, only robustly expressed genes were retained by requiring that the log (base 2) expression level be at least 7 for at least 2/3 of samples. We performed this filter for gene-level analysis because low- or un-expressed genes are noisy when examined individually. Next we normalized genes by subtracting the median value and dividing by the median absolute deviation. This scaling method is ideal because it is robust to outlier values. Two classes of outliers were then defined: high-outliers had expression values greater than the median plus the interquartile range (IQR), and low-outliers had expression values less than the median minus the IQR. Fisher’s exact tests were performed on contingency tables of high- or low-outliers by diagnosis, and the lower of the two p-values was kept. The Fisher’s exact tests were one-sided for ASD specificity. P-values were adjusted for multiple hypothesis testing using the q-value FDR estimation. Pathway enrichment was calculated for these outlier genes using hypergeometric tests on the MSigDB pathways.

Unless otherwise specified, all calculations were performed in R version 2.14. A complete R script is available from the authors upon request.

## Competing interests

The authors declare no conflicts of interest.

## Authors’ contributions

ISK and SWK conceived of the project; SWK and MGC designed the method; MGC performed the analysis; all authors contributed to writing and reviewing the manuscript. All authors read and approved the final manuscript.

## Pre-publication history

The pre-publication history for this paper can be accessed here:

http://www.biomedcentral.com/1755-8794/6/34/prepub

## Supplementary Material

Additional file 1 Results from propensity sampling, with p-values averaged over 100 trials. Click here for file

Additional file 2 **3D PCA of neuron development pathway in TGen.** The percent of variance captured by each principal component is shown. Click here for file

Additional file 3 Differentially expressed genes for each TGen subgroup. Click here for file

Additional file 4 Results from group difference methods (hypergeometric tests, GSEA) for TGen. Click here for file

Additional file 5 Overrepresented pathways from gene-level outlier analysis of TGen data set. Click here for file

Additional file 6 Outlier-enriched pathways (uncorrected p-value < 0.05) from the Simons data set. Click here for file

Additional file 7 Differentially expressed genes for the Simons neurogenesis subgroup. Click here for file

Additional file 8 Results from the Simons data set with McNemar’s test on sibling/proband pairs substituted for Fisher’s test. Click here for file

Additional file 9 Phenotype information for the TGen and Simons data sets. Click here for file

Additional file 10 **Microarray preprocessing.** TGen before (A) and after (B) removal of total outliers. Batch effects were not significant in TGen (C). Simons data set before (D) and after (E) ComBat and linear modeling. Click here for file
