# Supplementary figures and images for: Pathway-based outlier method reveals heterogeneous genomic structure of autism in blood transcriptome
Source: BMC Med Genomics. 2013 Sep 24;6:34. doi: 10.1186/1755-8794-6-34 (PMC3849321; doi:10.1186/1755-8794-6-34)

PCA of neuron development (TGen)

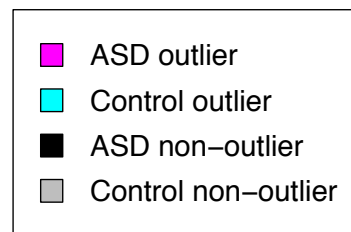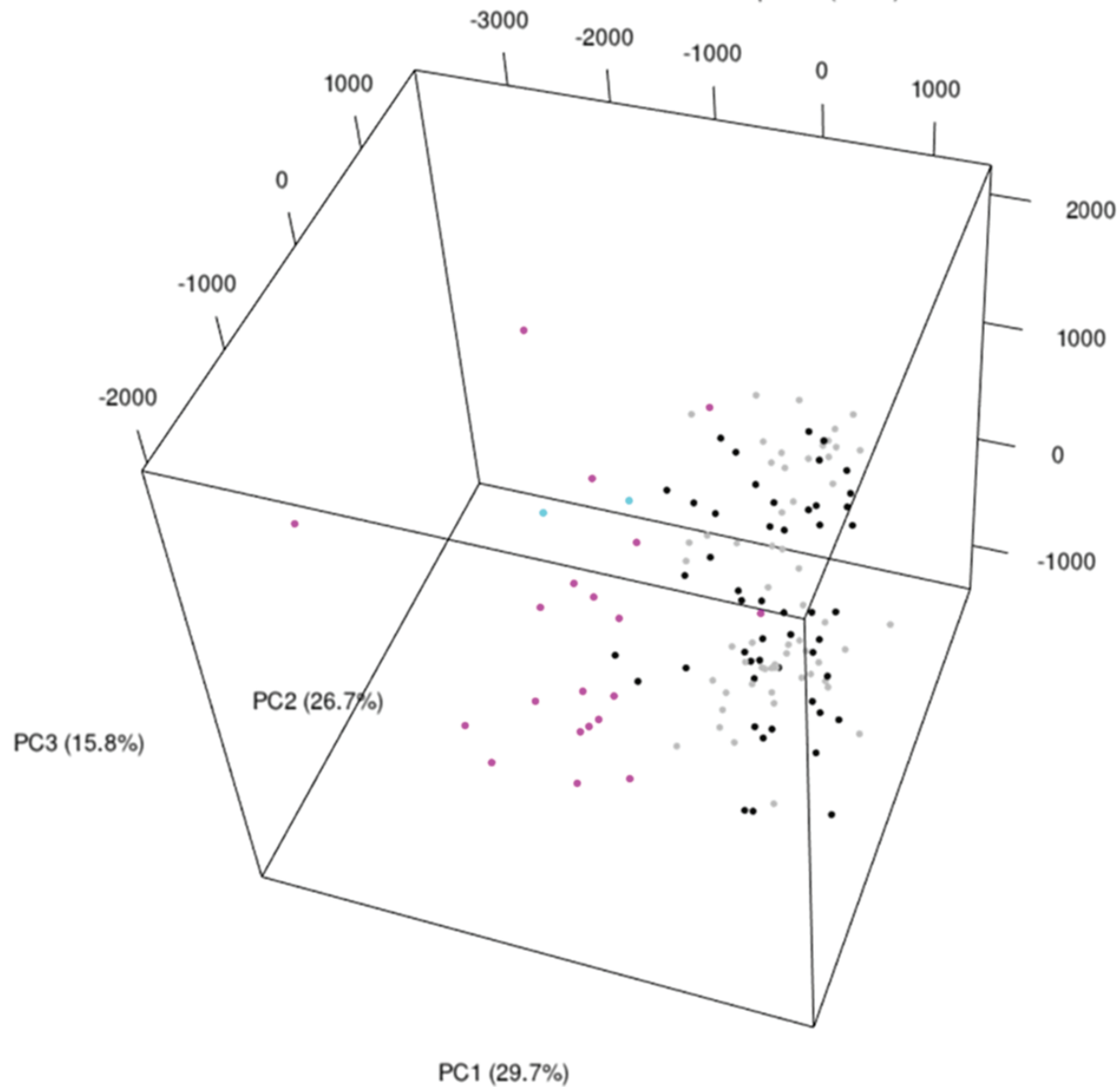

Supplement: Additional file 2 — 3D PCA of neuron development pathway in TGen. The percent of variance captured by each principal component is shown. [file 1755-8794-6-34-S2.pdf]

A.

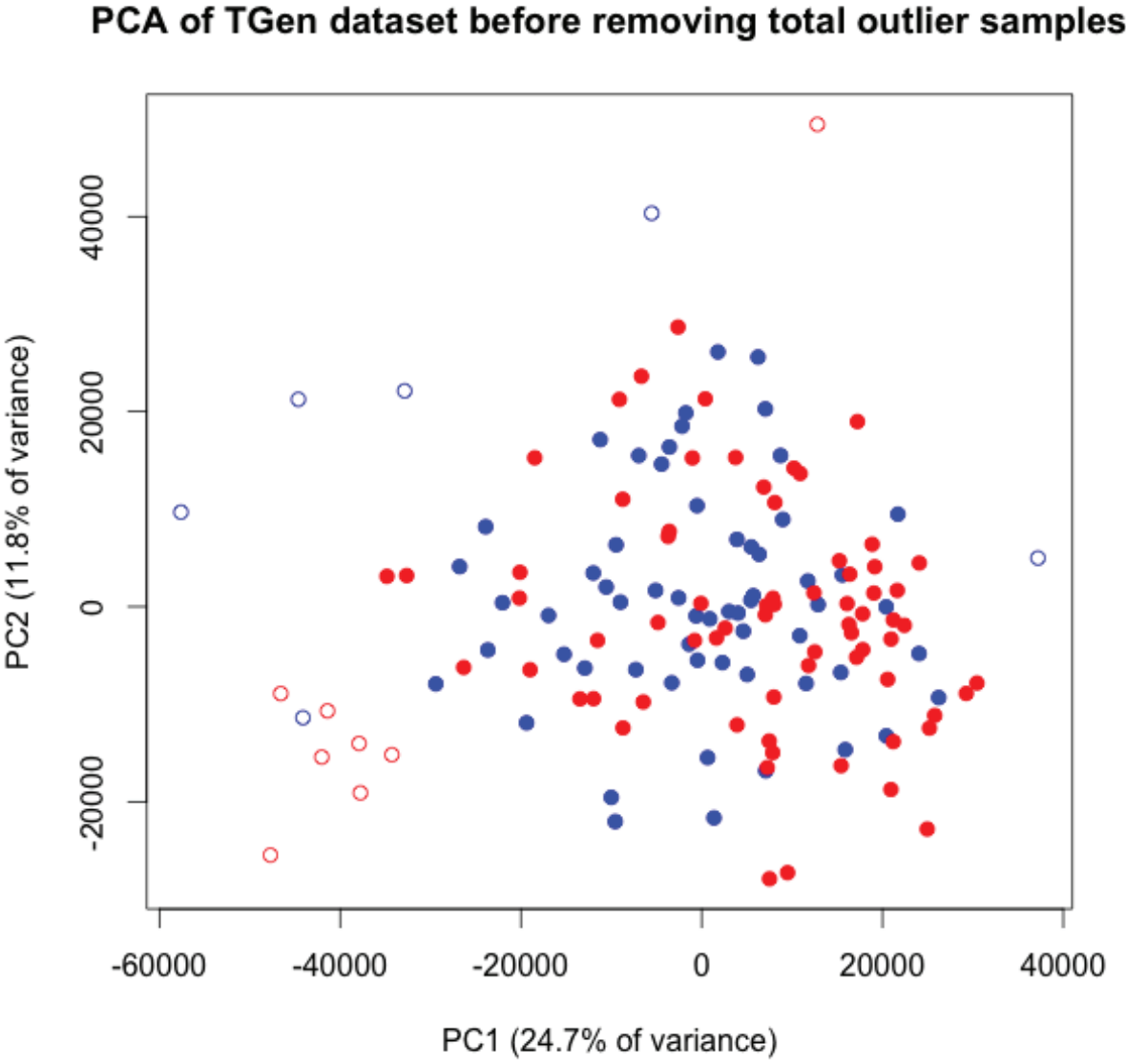

B.

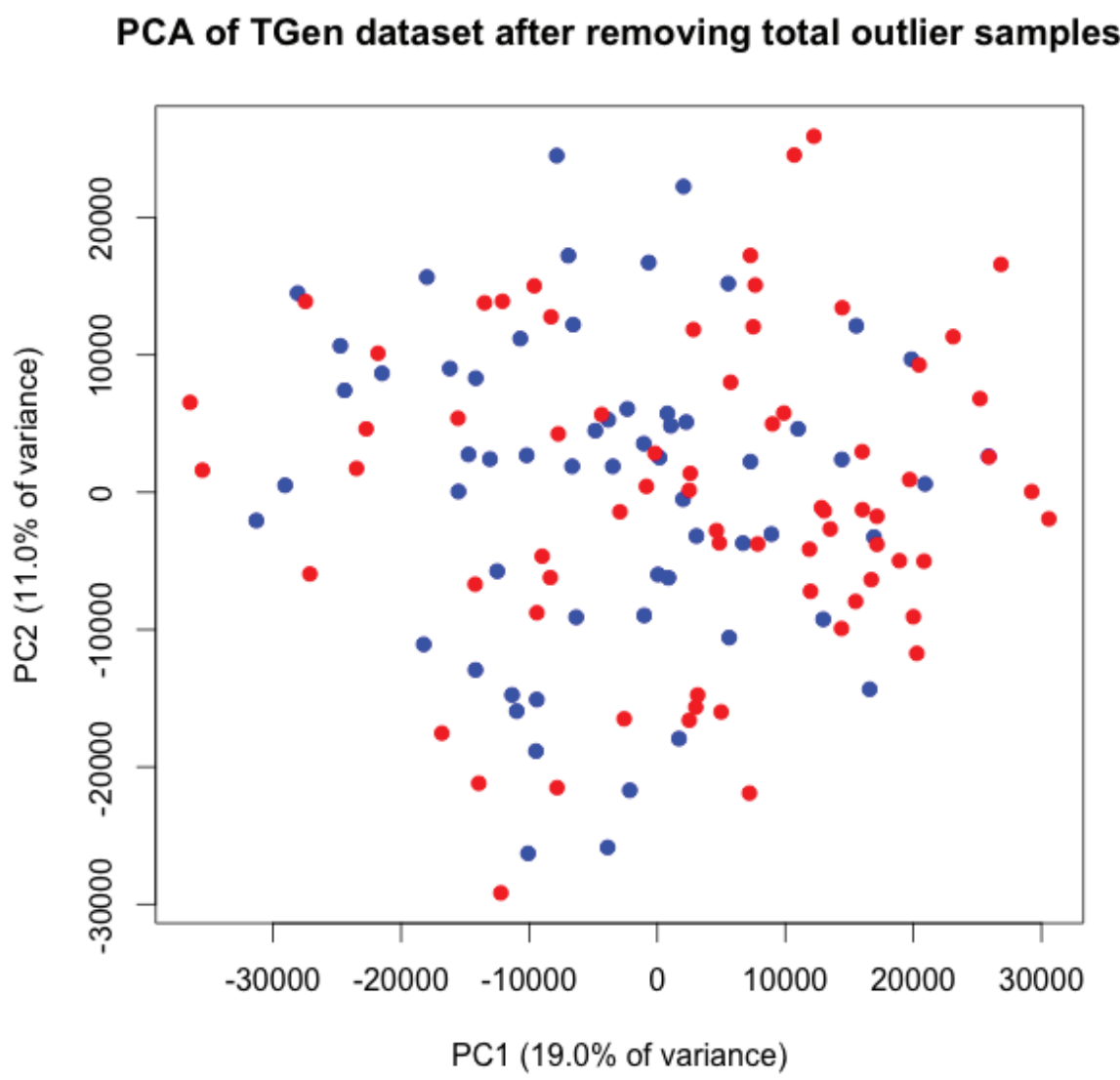

C.

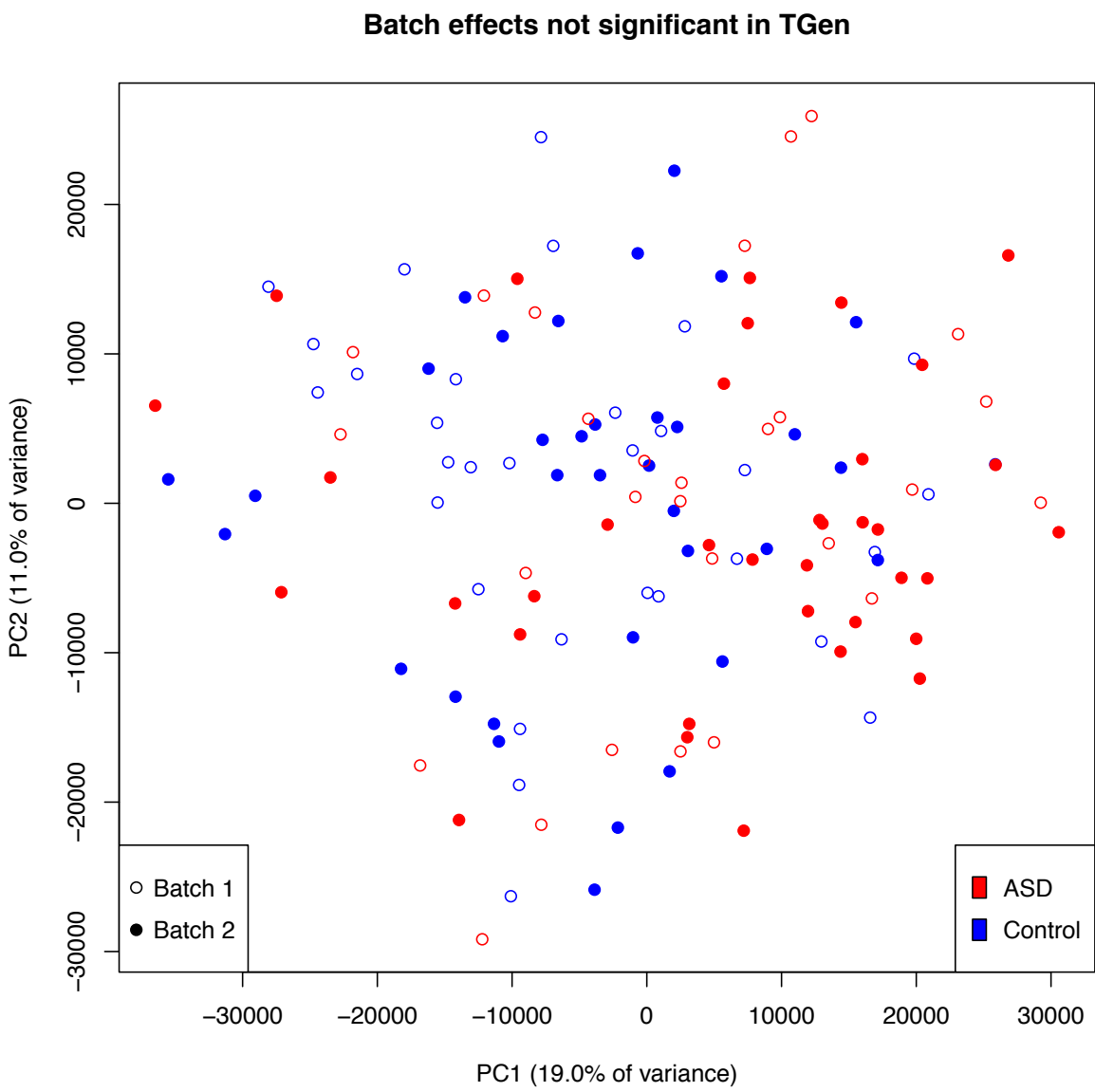

D.

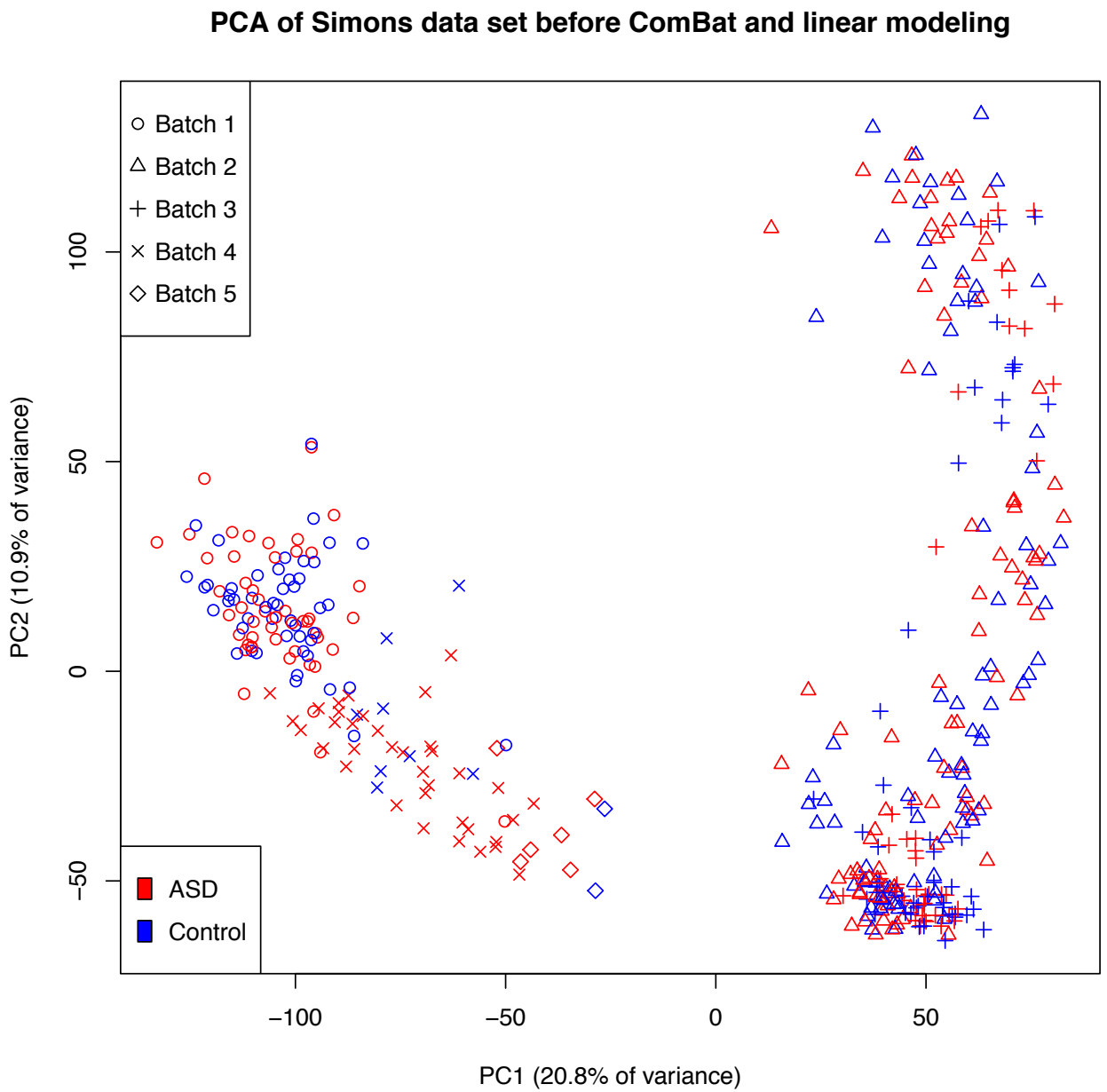

E.

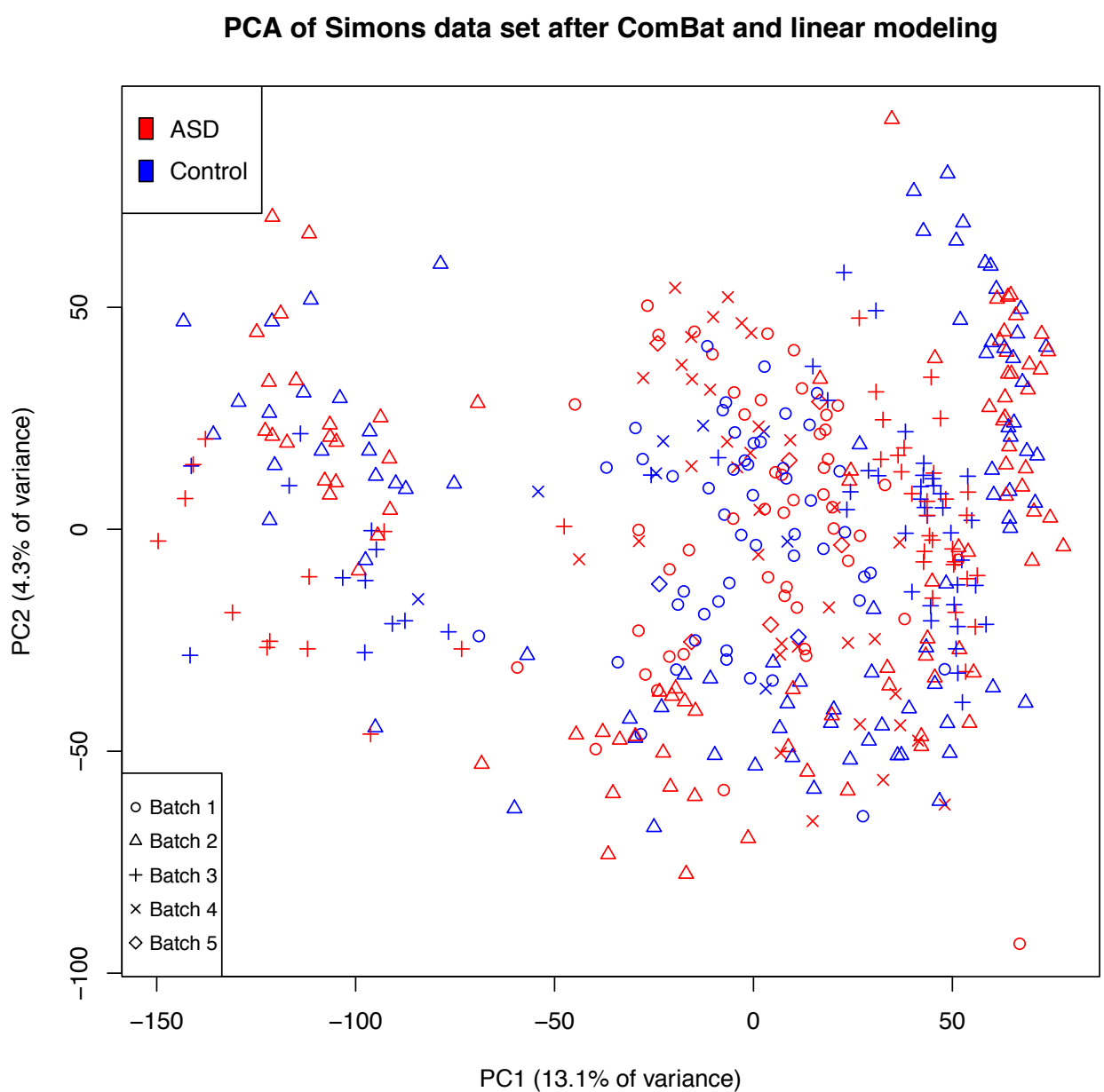

Supplement: Additional file 10 — Microarray preprocessing. TGen before (A) and after (B) removal of total outliers. Batch effects were not significant in TGen (C). Simons data set before (D) and after (E) ComBat and linear modeling. [file 1755-8794-6-34-S10.pdf]
