# Supplementary material for: Pathway-based outlier method reveals heterogeneous genomic structure of autism in blood transcriptome
Source: BMC Med Genomics. 2013 Sep 24;6:34. doi: 10.1186/1755-8794-6-34 (PMC3849321; doi:10.1186/1755-8794-6-34)
Supplement: Additional file 9 — Phenotype information for the TGen and Simons data sets. [file 1755-8794-6-34-S9.docx]

Additional file 9. Phenotype information for the TGen and Simons data sets. C = Caucasian, AA = African American, H = Hispanic, AS = Asian, M = Mixed, U = Unknown. Ages are shown as range, mean, median.

|  | **TGen** | | | | **Simons** | |
| --- | --- | --- | --- | --- | --- | --- |
|  | **Case** | **Control** | | | **Case** | **Control** |
| Total | 70 | 60 | | | 221 | 191 |
| Race/Ethnic group | 46C, 1AA, 5H, 12M, 6U | | 34C, 1AS, 9M, 16U | | 159C, 11AS, 7AA, 44U | 138C, 11AS, 6AA, 36U |
| Age | 2.7-11.7, 6.1, 5.7 | | | 2.4-11.4, 8.5, 9.3 | 4.0-17.7, 9.1, 9.6 | 3.8-41.8, 9.5, 10.5 |
| Sex | All M | | | All M | 188 M, 33 F | 100 M, 91 F |
| Batch | 30 batch 1, 40 batch 2 | | | 30 batch 1, 30 batch 2 | 49 batch 1, 88 batch 2, 42 batch 3, 36 batch 4, 6 batch 5 | 49 batch 1, 83 batch 2, 49 batch 3, 8 batch 4, 2 batch 5 |
